# Polylactic‐ Glycolic Acid Microparticles–Encapsulated Prostaglandin E1 as A Novel Strategy In Triple Negative Breast Cancer

**DOI:** 10.1002/open.202500364

**Published:** 2025-09-19

**Authors:** Concetta Di Natale, Elena Lagreca, Raffaele Crispino, Roberta D’Auria, Alessandro Attanasio, Rezvan Jamaledin, Raffaele Vecchione, Paolo Antonio Netti

**Affiliations:** ^1^ Dipartimento di Ingegneria Chimica dei Materiali e della Produzione Industriale University of Naples Federico II P. le Tecchio 80 Napoli I‐80125 Italy; ^2^ Department of Life Science University of Bath Claverton Down Bath BA2 7AY UK; ^3^ Interdisciplinary Research Centre on Biomaterials (CRIB) University of Naples Federico II P. le Tecchio 80 Naples 80125 Italy

**Keywords:** cell cytotoxicities, drug deliveries, polylactic‐ glycolic acid microparticles, prostaglandins E_1_, triple breast cancers

## Abstract

Triple‐negative breast cancer (TNBC) is characterized by unique clinical and pathological traits, from which its aggressive nature and the absence of specific treatments are the most worrying. Prostaglandin E_1_ (PGE_1_) can affect the morphology of human breast tumor cell lines, but its potential therapeutic effects appear to be counteracted by its high degradability in physiological environments. For this reason, polylactic‐glycolic acid polymeric microparticles (MPs) are developed to stabilize PGE_1_ in damp conditions and enable sustained local release for the treatment of TNBC after surgical removal of the tumor mass. Specifically, the PGE_1_ embedded MPs are produced using a double emulsion solvent‐evaporation method, then analyzed through Mastersizer and scanning electron microscopy. Afterward, the encapsulation efficiency and the PGE_1_ release are examined using liquid chromatography–mass spectrometry, and their stability at various storage temperatures is assessed. Finally, the carrier toxicity is evaluated in TNBC cells and colon adenocarcinoma epithelial cells, Caco‐2. A reliable action on carcinogenic cells specific to breast cancer is observed. Although in vivo studies are still needed, these results open the way to using such a carrier for the intralesional delivery of PGE_1_ and its use against TNBC.

## Graphical abstract

Schematic representation of the production of prostaglandin E_1_‐loaded microparticles through a solvent evaporation double emulsion technique, followed by their physicochemical characterization and in vitro assessment in breast cancer cells.

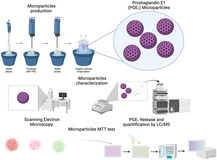

## 1 Introduction

Triple‐negative breast cancer (TNBC) is characterized by the absence of estrogen and progesterone receptors, often displaying aggressive behavior with high relapse rates and metastatic potential.^[^ ^1^ ^]^ Although in recent years there has been an increase in patients surviving, primarily due to improvements in screening methods, the surgical approach and poly‐chemotherapy are still the standard treatments despite their related serious toxic effects, resulting in patients having a median overall survival of only a few months.^[^ ^2^ ^]^ Surgical interventions, such as lumpectomy or mastectomy with sentinel lymph node biopsy or axillary dissection, are often followed by conventional chemotherapy or radiation therapy to eradicate residual tumor cells.^[^ ^3^ ^]^ However, these approaches are frequently associated with systemic toxicity and limited efficacy in preventing recurrence. To overcome these limitations, localized drug delivery systems for intralesional administration, are emerging as promising alternatives to overcome these limitations.^[^ ^4^, ^5^ ^–^ ^6^ ^]^ By enabling sustained release of therapeutics directly at the tumor site, they minimize systemic exposure while improving treatment efficacy.^[^ ^7^ ^]^ This strategy is under investigation in preclinical and clinical settings, particularly for administering monoclonal antibodies, cellular therapies, immune agonists, and other immunomodulatory agents.^[^ ^8^ ^]^ In this direction, an improved strategy is based on surgical intervention, combined when possible, with postsurgical intralesional administration, that is, the direct injection of therapeutics into the postsurgical tumor bed, promising enhanced local drug concentrations, reducing off‐target effects, and improving locoregional disease control.^[^ ^9^, ^10^, ^11^ ^–^ ^12^ ^]^ Nonetheless, challenges persist, including poor retention of small‐molecule drugs within the tumor microenvironment and rapid clearance, which may limit therapeutic efficacy. In this context, microparticle (MP)‐based systems represent a valuable technological advancement. For instance, developing localized, biocompatible drug delivery systems such as polymeric MPs provided promising results in the postsurgical management of TNBC, which remains a clinical challenge.^[^ ^13^ ^]^ In this context, large‐surface‐area MPs, like those developed by our research group, offer the dual advantage of sustained drug release and improved local retention.^[^ ^14^ ^]^ Previous studies with albumin microspheres and paclitaxel‐loaded MPs have demonstrated enhanced tumor accumulation and superior antitumour activity compared to systemic delivery routes.^[^ ^15^ ^]^

Despite its potential, local drug delivery faces challenges such as poor drug retention and resistance due to limited chemotherapy levels within the tumor. Large‐surface‐area MPs, like those developed by our research team, enable sustained drug release and improved tumor retention.^[^ ^16^ ^,^ ^17^ ^]^ Studies show that albumin microspheres and paclitaxel‐loaded MPs significantly enhance local drug concentrations and antitumor efficacy compared to intravenous administration.^[^ ^18^ ^]^ Recently, a new class of drugs known as prostaglandins E_1_, particularly (PGE_1_), have emerged as a promising agent in cancer treatment due to their ability to inhibit tumor growth and metastasis.^[^ ^19^ ^]^ Specifically, PGE_1_ shows selective cytotoxic effects on human breast cancer cell lines like MDA‐MB‐231 and MCF‐7.^[^ ^4^ ^,^ ^20^ ^,^ ^21^ ^]^ Beyond cancer therapy, PGE_1_ has potential in reconstructive surgery: a study by Hwang et al. demonstrated that PGE_1_ treatment reduced skin complications, wound revision rates, and nipple necrosis in implant‐based breast reconstruction after mastectomy, suggesting its role as a protective adjuvant therapy.^[^ ^22^ ^]^ Despite its therapeutic employment, fast metabolization and deactivation of PGE_1_ in plasma require long‐term infusions to maintain effective treatment concentrations, often leading to severe side effects such as systemic allergic reactions, hypotension, peripheral edema, or hemorrhage.^[^ ^23^ ^]^ Various PGE_1_ delivery systems have been developed to address this challenge, including lipid‐based nano/microspheres, cyclodextrin‐polymer conjugates, and soybean oil‐based emulsions to improve molecule stability and modulate pharmacokinetics.^[^ ^24^ ^]^ However, these systems still have limitations, such as chemical instability, rapid drug leakage in the bloodstream, and persistent local side effects.^[^ ^24^ ^,^ ^25^ ^]^ Our group has pioneered the encapsulation of biomolecules using super‐porous polylactic‐glycolic acid (PLGA)MPs to enhance chemical and biological stability.^[^ ^17^ ^,^ ^26^ ^,^ ^27^ ^]^ Previously, we successfully incorporated collagenase into PLGA MPs, achieving protection against inactivation under storage conditions and tunable drug release. We previously demonstrated successful PLGA incorporation of collagenase, achieving i) protection from inactivation due to storage conditions such as temperature and pH, ensuring enzyme activity of almost 100 nkat and ii) tunable drug release for prolonged therapeutic effects. Building on this expertise, we have developed highly porous PLGA MPs encapsulating PGE_1_ to provide sustained drug release within a few hours, addressing the molecule's inherent instability in aqueous environments while enhancing its pharmacological activity and minimizing toxic side effects.^[^ ^14^ ^]^ Building on this expertise, we have developed highly porous PLGA MPs encapsulating PGE_1_, enabling sustained drug release over several hours. This formulation enhances PGE_1_ stability in aqueous environments, prolongs its pharmacological activity, and minimizes toxic side effects. Our platform holds potential as a localized treatment for TNBC through intralesional administration following surgical tumor resection, ensuring high local drug concentrations while avoiding systemic complications. This study demonstrates the stability, loading efficiency, and controlled release of PGE_1_ within PLGA MPs. Furthermore, in vitro evaluations confirm selective cytotoxicity against MDA‐MB‐231 tumor cells while sparing noncancerous MCF10A cells, supporting its potential as a targeted and effective TNBC therapy.

## 2 Experimental Section

### 2.1 MPs Synthesis and Characterization

**Figure 1:**
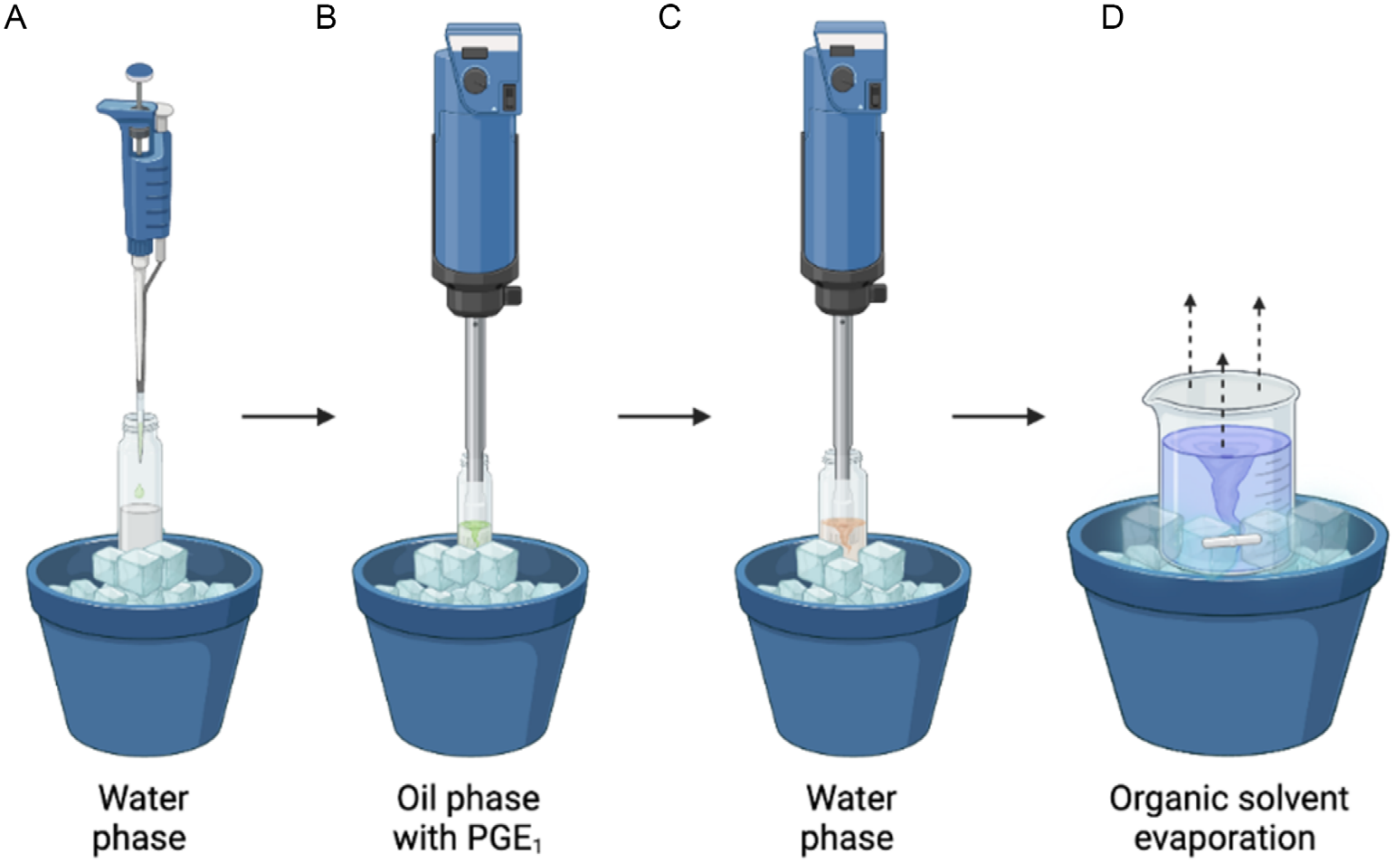
Schematic representation of double emulsion water/oil/water (w/o/w) double emulsion/solvent evaporation technique for PGE_1_–MP production.

PGE_1_–MPs were obtained using the previously described water/oil/water (w/o/w) double emulsion/solvent evaporation technique, adding a stabilizing agent, the Pluronic F‐68. As reported, PGE_1_ MPs were prepared using the w/o/w double emulsion/solvent evaporation technique^[^ ^14^ ^,^ ^17^ ^,^ ^26^ ^–^ ^28^ ^]^ (**Figure** **1**). The oil phase consisted of 10 mg of PGE_1_ dissolved in 100 μL of ethanol (EtOH) and added to a dichloromethane (DCM) solution in which 83 mg mL^−1^ of PLGA and 17 mg mL^−1^ of Pluronic F‐68 were previously dissolved. The aqueous phase, consisting of 100 μL of bidistilled water, was homogenized (Ultra‐turrax, IKA T‐25 ULTRA‐TURRAX Digital High‐Speed Homogenizer Systems) for 30 s at 15,000 rpm with the oil phase. This first emulsion was immediately poured into 10 mL of 2% (w/v) polyvinyl alcohol (PVA) solution and homogenized for 1 min at 20,000 rpm. To allow DCM evaporation, the second emulsion was finally poured into 40 ml of water under mechanical stirring for 3 h at 450 rpm with a paddle stirrer (Heidolph RZR 2102 control). Then, the MPs were collected and washed three times with Milli‐Q water by centrifuging at 10,000 rpm for 10 min at 4 °C (SL16R Centrifuge, Thermoscientific, USA), lyophilized overnight (HetoPowerDry PL6000 Freeze Dryer, Thermo Electron Corp., USA; −50 °C, 0.73 hPa), and stored at −20 °C with desiccating agents until further investigation. All the steps were performed by keeping the samples in an ice bath to preserve the PGE_1_ stability during the procedure.

The morphological analysis was performed using scanning electron microscopy (SEM), following the procedure previously described.^[^ ^29^ ^,^ ^30^ ^]^ Briefly, a 20 µL sample was deposited on a standard SEM pin stub and examined using a field emission scanning electron microscope (FESEM) (FESEM ULTRA‐PLUS, Zeiss, Milan, Italy) and then placed into a sputter coater, which covered them with a 15 nm thickness gold layer. Furthermore, the internal porous structure of the MPs was investigated using a polydimethylsiloxane (PDMS) method.^[^ ^31^ ^]^ A PDMS layer, 2 mm in thickness, was cured at 80 °C for 30 min. After cooling, the MPs were deposited on this layer, and another 2 mm‐thick PDMS layer was used to cover them. The resulting solid PDMS block was frozen in liquid nitrogen at –196 °C and sectioned using the Leica CryoUltra Microtome EM‐FC7‐UC7 (Milan, Italy). The imaging was conducted at an accelerating voltage of 5 kV with the SE2 detector. The MP size and polydispersity index were measured using static light scattering using the Mastersizer 3000 instrument from Malvern Instruments (Malvern, UK). The PGE_1_–MPs were dispersed in water at 3 mg mL^−1^ for these measurements.

### 2.2 PGE_1_ Encapsulation Efficacy (%n) and In Vitro Release by Liquid Chromatography–Mass Spectrometry (LC–MS)

To determine the total amount of PGE_1_ encapsulated in MPs, 5 mg of MPs were dissolved in 1 mL of DMSO using a gentle magnetic stirring (350 rpm) for 15 min. Afterward, the solution was centrifuged for 5 min at 10,000 rpm, and the supernatant was analyzed by Agilent 6530 Accurate‐Mass‐quadrupole time‐of‐flight ultra‐performance LC (UPLC)/MS system employing UPLC for PGE_1_ fractionation and MS for analyte detection and quantification. A Zorbax RRHD Eclipse Plus C18, 2.1 × 50 mm, 1.8 µm columns were used for these analyses, applying a linear gradient of 0.1% (v/v) formic acid in acetonitrile (CH_3_CN)/0.1% formic acid in water from 50% (v/v) to 70% (v/v) over 20 min at a flow rate of 0.1 µL min^−^^1^. In detail, the peak of PGE_1_ (protonated mass = 389.22 m z^−1^) was identified (Figure S1‐A, Supporting Information), and its area under the curve (AUC) was measured for a quantitative analysis using the titration curve of the free PGE_1_ dissolved in DMSO at concentrations from 0 to 600 mg mL^−1^ (Figure S1‐B, Supporting Information). The release was done by dissolving 10 mg of PGE_1_–MPs in 1.5 mL of phosphate saline buffer (PBS). PGE_1_–MPs were incubated at 37 °C and shaken at 350 rpm. At a fixed period (15 min to 120 h), 1 mL of sample was withdrawn from the supernatant during MP sedimentation by using centrifugation for 15 min at 10,000 rpm (MicroCL21R, Centrifuge, Thermoscientific, USA) and analyzed by LC–MS, as previously reported. The sedimented MPs were resuspended in the same volume of fresh buffer. A titration curve of free PGE_1_ dissolved in PBS was performed for LC–MS analysis and quantification (data not shown). All the tests were performed in triplicate, and the % average for the release was calculated as

(1) $$\%\text{Release}=\left(\frac{\text{actual drug loading}}{\text{theoretical drug loading}}\right)*100\%$$

where the theoretical drug loading is the concentration of drug that should have been loaded, while the actual drug loading is the real concentration of the drug that was found.

### 2.3 PGE_1_–MPs Stability

The stability of embedded PGE_1_ was quantified by LC–MS using the titration curve in DMSO (Figure S1, Supporting Information). Three batches of PGE_1_‐MPs stored at 4 °C or room temperature were dissolved in DMSO, and the AUC of the mass (peak 389.22 m z^−1^) was identified and quantified at 0 until 30 d of storage. The same experiments were performed for free PGE_1_ in the same storage conditions. Data were normalized compared to the protein concentration and the weight of MPs and expressed as a percentage.

### 2.4 Cell Culture Preparation

MDA‐MB‐231 cells (generously provided by Francesco Paolo Cammarata, Institute of Molecular Bioimaging and Physiology, National Research Council IBFM‐CNR, 90,015 Cefalù, Italy) were cultured in Dulbecco's Modified Eagle's Medium (DMEM)/Nutrient Mixture F‐12 Ham (Sigma, St. Louis, MO, USA) supplemented with 10% fetal bovine serum(FBS) (FBS, Gibco, Germany), 1% L‐glutamine (Sigma, USA), and 1% penicillin–streptomycin (Sigma, St. Louis, MO, USA).

MCF10A cells (generously provided by Stefano Piccolo, AIRC Institute of Molecular Oncology, 20,139 Milan, Italy) were cultured in the same basal medium but supplemented with 5% horse serum, 1% L‐glutamine (Sigma, USA), 1% penicillin–streptomycin (Sigma, USA), 0.1% epithelial growth factor, 0.1% insulin, and 0.1% hydrocortisone.

Human epithelial colorectal adenocarcinoma cell lines (Caco‐2) were purchased from the American Type Culture Collection (ATCC) and cultured in high‐glucose DMEM (DMEM Sigma, USA) with the addition of 10% FBS (FBS, Gibco, Germany), 1% L‐glutamine (Sigma, USA), and 1% penicillin–streptomycin (Sigma, St. Louis, MO, USA).

All human cell lines have been authenticated using short tandem repeat profiling within the last 3 years and are listed using the official cell line name.

#### 2.4.1 Cell Viability

TNBC (MDA‐MB‐231), mammary gland epithelial cells (MCF10A), and a colon carcinoma‐associated cell line (Caco‐2) were seeded in triplicate in 96‐well plates at a 12,500 cells/well density and allowed to adhere overnight. Empty MPs and PGE1–MPs were diluted in cell culture medium at final concentrations of 1.5 mg ml^−1^ and added to the cells for 24, 48, and 72 h. After the incubation, the MTT cell growth assay, a colourimetry assay used for either proliferation or complement‐mediated cytotoxicity assays, was performed according to the manufacturer's instructions. Briefly, MTT powder (thiazolyl blue tetrazolium bromide, Sigma, USA) was dissolved in the cell medium at a final concentration of 0.5 mg mL^−1^ and filtered to avoid possible particulates. Following PBS washing to eliminate the particles from the wells, this solution was put in contact with the cells for 3 h at 37 °C in a humidified incubator. Once the supernatant was removed, 200 µL of 2‐propanol was used in each well to dissolve MTT crystals and left to act for 20 min. Then, a plate reader (Enspire plate reader, Perkin Elmer, USA) was used at 570 nm. The emission of each well was compared to the control to obtain the viability percentage.

## 3 Results and Discussion

### 3.1 Preparation and Structural/Morphological Characterization of PGE_1_–MPs

**Figure 2:**
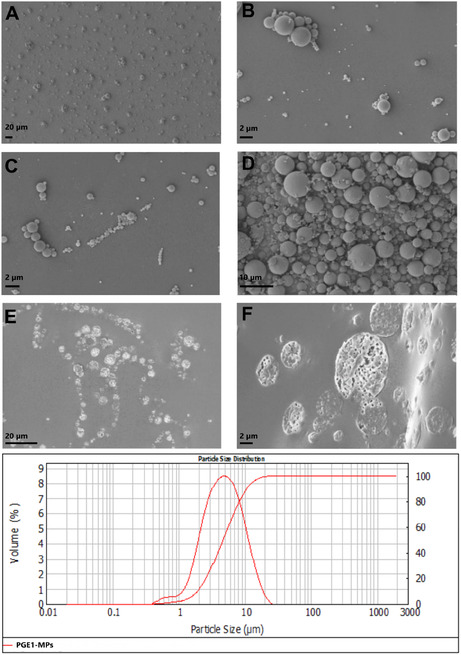
A–D) Top PGE_1_–MPs SEM images, E–F) PGE_1_–MPs cryosection embedded in PDMS. Bottom PGE_1_–MPs Mastersizer size distribution.

The PGE_1_–MPs were prepared using the double emulsion solvent‐evaporation technique. Following washing steps and lyophilization, the particles were suspended, and their size was assessed using a laser diffraction particle size analyzer. The size distribution showed a good value with a mean diameter of 5.0 ± 0.2 µm (**Figure** **2A**). SEM also evaluated a deeper characterization to confirm the MPs uniformity in distribution and size. As shown in Figure 2B–E, MPs were predominantly spherical and mostly isolated from one another, corroborating the Mastersizer analysis. Moreover, the SEM images highlighted MPs with a nonporous external surface (Figure 2B–E) with an evident inner porous configuration, as shown in the internal section in Figure 2F–G, acquired using the PDMS method.^[^ ^25^ ^]^ The morphology of the MPs, particularly their smooth surface and internal porosity, is influenced by the presence of EtOH in the oil phase. Being a solvent, EtOH positively affects MP surface structure and internal porosity when adequately balanced. Specifically, increasing the concentration of EtOH in the formulation leads to a decrease in porosity, with the surface showing more imperfections and becoming less smooth.^[^ ^32^ ^,^ ^33^ ^]^

### 3.2 PGE_1_–MP Entrapment Efficiency and Release in PBS to Mimic Physiological Conditions

**Figure 3:**
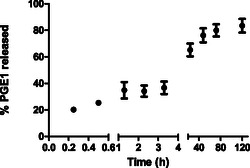
%PGE_1_ in vitro release in PBS pH 7.2 from 0 to 120 h.

The entrapment efficiency of PGE_1_–MPs was evaluated by LC–MS as described in the Experimental Section. Specifically, an encapsulation efficiency of 29.3 ± 4.1% was obtained by analyzing the AUC of the PGE_1_ peak (protonated mass = 389.22 m z^−1^) and performing a quantitative analysis using the titration curve of free PGE_1_ dissolved in DMSO at concentrations from 0 to 600 mg mL^−1^ (Figure S1 A‐B, Supporting Information). Regarding the in vitro release of PGE_1_ from MPs, an initial burst phase was observed from 0.2 to 0.5 h (**Figure** **3**, **Table** **1**), followed by a stationary phase from 1 to 4 h (Figure 3, Table 1) and then an extended exponential phase from 40 to 120 h, in which over 80% of the molecule was released (Figure 3, Table 1). These results are consistent with the morphological features of the obtained MPs. Pluronic facilitated the formation of a well‐crosslinked and internally porous structure, which is crucial for controlled drug release. Given PGE_1_ sensitivity to physiological and environmental factors such as temperature, humidity and pH, a controlled release that is not overly prolonged is desirable. The nonporous external surface shields the molecule from moisture, allowing it to remain intact for around 72 h. LC–MS analysis at 120 h revealed the degradation onset, with the PG A_2_ (PGA_2_) byproduct formation, as depicted in Figure S2, Supporting Information.

**Table 1:** Quantification of % PGE_1_ in vitro release in PBS pH 7.2 from 0 to 120 h.

Time [h]	[%] of PGE_1_ released in PBS
0.25	20.2 ± 1.5
0.5	25.4 ± 1.7
1.30	34.9 ± 6.
2.30	34.2 ± 4.2
3.30	36.7 ± 4.7
24	65.2 ± 4.9
48	76.2 ± 5.3
72	79.9 ± 4.6
120	83.4 ± 5.1

By comparing our developed formulations with state‐of‐the‐art PLGA MPs for PGE_1_ delivery, we demonstrate improved molecular stability without relying on cyclodextrin complexes commonly used in other PGE_1_ formulations. Notably, the PGE_1_‐hydroxypropyl‐β‐cyclodextrin (HP‐β‐CD) inclusion complex, while enhancing solubility, has significant limitations, including i) reduced drug entrapment efficiency due to increased water solubility, which makes the complex more prone to leaching from the MP core and ii) increased particle size and bulk associated with HP‐β‐CD incorporation.^[^ ^34^ ^]^ Despite the absence of a porous structure on the surface of our PLGA MPs, the drug release kinetics remain effective, displaying the characteristic biphasic profile typical of PLGA MPs, with an initial burst followed by sustained release. While formulations based on PGE_1_‐HP‐β‐CD inclusion complexes ensure surface porosity due to the presence of HP‐β‐CD, this approach often presents drawbacks, including reduced drug entrapment efficiency, caused by increased water solubility of the complex, and increased particle bulk. Our strategy, in contrast, avoids these drawbacks, demonstrating that porosity is not a prerequisite for achieving optimal release kinetics in PLGA‐based systems. Unlike previous studies utilizing inhalable PLGA MPs for PGE_1_ delivery in pulmonary hypertension models, our nonporous PLGA MPs, designed for intralesional administration in TNBC therapy, demonstrated enhanced drug encapsulation efficiency and stability without relying on inhalation‐based approaches. Moreover, while Ghosh et al. achieved sustained drug release through a pulmonary route, our formulation maintains a controlled biphasic release profile suitable for localized cancer treatment, highlighting the versatility of nonporous PLGA MPs across different therapeutic applications.

### 3.3 PGE_1_–MP Stability at Different Storage Conditions

**Figure 4:**
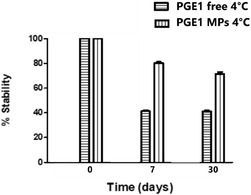
Stability test of PGE_1_–MPs and free molecule at 4 °C.

Numerous studies have underscored the inherent instability of PGE_1_ under physiological conditions, particularly at neutral pH and elevated temperatures. PGE_1_ demonstrates significantly greater stability in mildly acidic saline solutions (pH 4.5–4.7), retaining ≈25% of its initial concentration after 32 d at 37 °C, whereas degradation exceeds 95% under neutral conditions (pH 7.4) within just two weeks. Similarly, PGE_1_ stored at 4 °C in EtOH saline mixtures exhibited a markedly prolonged shelf life exceeding 100 d, compared to a stability of less than 10 d at room temperature.^[^ ^35^ ^]^ Improved stability has also been achieved through specific formulation strategies, including storage in light‐protected polypropylene syringes and complexation with cyclodextrins, especially HP‐β‐CD, which enhances both solubility and chemical stability.^[^ ^36^ ^]^ These findings highlight the critical need for advanced delivery systems to preserve the pharmacological activity of PGE_1_ and support its clinical use in chronic therapies.^[^ ^34^ ^,^ ^36^ ^,^ ^37^ ^]^ To assess the stabilizing efficacy of our system, PGE_1_‐loaded PLGA MPs were stored at 4 °C. The storage at 4 °C is preferable, as it is a standard condition for thermolabile compounds, minimizing enzymatic degradation and microbial proliferation. As shown in **Figure** **4**, the polymeric matrix provided significant protection: while the free molecule lost ≈60% of its activity within just 7 d at 4 °C, the encapsulated form retained 80% of its initial content after 30 d. These results highlight the robust protective capacity of our PLGA‐based system, ensuring PGE_1_ stability even under refrigerated conditions, which is an essential requirement for regulatory approval and clinical application.

### 3.4 Cytotoxicity

**Figure 5:**
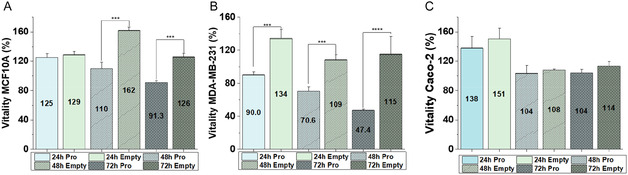
MTT vitality test for empty and PG‐loaded MPs at different time points (24 h, 48 h, and 72 h). The tests were performed on different cell lines: A) healthy mammary cells, MCF10A; B) triple‐negative human breast adenocarcinoma cells, MDA‐MB‐231; and C) tumor colonic epithelial cells, and Caco‐2. (***p‐value < 0.001; ****p‐value < 0.0001).

To demonstrate the efficacy of our system on mammary tumors, an MTT test was performed on three cell lines: the TNBC (MDA‐MB‐231), mammary gland epithelial cells (MCF10A), and a colon carcinoma‐associated cell line (Caco‐2), to assess the specificity of the carrier. The test included empty particles as a control. Notably, the particles did not affect the vitality of healthy mammary cells (**Figure** **5A**), with values exceeding 100% in each case, except for the 72 h contact with PGE_1_–MPs, where cell viability was around 91%, still comparable with the control. A similar trend was observed for Caco‐2 cells (Figure 5C), where viability remained above 100% under all conditions. The fact that cell viability was above 100% indicates that the cells experienced heightened metabolism. The MTT assay reflects cellular respiration and can be influenced by compounds interfering with energy metabolism, leading to values above 100%.^[^ ^38^ ^,^ ^39^ ^]^ Furthermore, PLGA is usually degraded by cells through the Krebs cycle, potentially enhancing cell metabolism and explaining the high vitality level.^[^ ^40^ ^]^ On the other hand, there is a consistent difference when considering tumor mammary cells (Figure 5B). In this case, the effect of PGE_1_–MPs is evident, with cell viability reduced to about 47% after 72 h of contact. This suggests that the MPs specifically target tumor mammary cells, as healthy cells and other tumor cells are poorly affected by their presence in the medium. This finding opens the possibility of using this carrier to fight breast cancer.

## 4 Conclusions

Although there is increasing attention to finding new treatments, TNBC remains a highly aggressive tumor with a poor response to traditional chemotherapy. In this context, our research may introduce a new method using a novel category of molecules: PG, especially PGE_1_. This molecule was found to act on MDA‐MB‐231 cells, but its therapeutic effects are often limited due to its high instability in moist physiological conditions. Herein, we have developed a novel drug delivery system involving porous PLGA MPs, known for their effectiveness in encapsulating and maintaining the molecular properties. Our system demonstrates enhanced stability, controlled drug release, and selective cytotoxicity toward TNBC cells, providing a foundation for further preclinical and clinical investigations. In detail, encapsulated PGE_1_ mainly shows 80% stability at 4 °C for 30 d, which is the typical drug storage condition, whereas the free form only maintains 40% stability under the same conditions. Moreover, our system has demonstrated specific toxicity toward MDA‐MB‐231 cells compared to control cells (MCF10A) and the Caco‐2 cell line. These initial findings pave the way for a novel and targeted treatment strategy for TNBC. Ultimately, by achieving high stability, PGE_1_ can be administered in quantities that do not harm healthy cells while providing therapeutic benefits. Here, the possibility of modeling the drug kinetics is of great help, and we will endeavor to include it in future studies to elucidate better the amounts delivered.^[^ ^17^ ^,^ ^41^ ^]^ We recommend that a local administration exploit the different roles of PGE_1_ in MDA‐MB‐231 cells. This approach could significantly improve patient outcomes, reduce recurrence rates, and offer a safer alternative to conventional treatments by achieving high local drug retention and reducing systemic exposure. Future studies will focus on optimizing formulation parameters and validating the in vivo efficacy of this localized delivery strategy, paving the way for its potential clinical translation in TNBC therapy.

## Conflict of Interest

The authors declare no conflict of interest.

## Author Contributions

**Concetta Di Natale** and **Elena Lagreca**: performed all the experiments and data analysis, wrote the draft and the final version of the manuscript with **Raffaele Vecchione** and **Raffaele Crispino** performed cellular analysis, wrote the corresponding paragraph and revised the manuscript. **Rezvan Jamaledin**, **Alessandro Attanasio**, and **Roberta D’Auria** helped in microparticle synthesis and characterization. **Raffaele Vecchione**, **Concetta Di Natale**, and **Paolo Antonio Netti** designed the concept, supervised the experiments, and discussed the results. All authors contributed to the drafting of the final version of the manuscript. **Concetta Di Natale** and **Elena Lagreca** contributed equally to this work.

## Supporting information

Supplementary Material

## Data Availability

The data that support the findings of this study are available from the corresponding author upon reasonable request.
