# Supplementary material for: Polylactic‐ Glycolic Acid Microparticles–Encapsulated Prostaglandin E1 as A Novel Strategy In Triple Negative Breast Cancer
Source: ChemistryOpen. 2025 Sep 19;14(12):e202500364. doi: 10.1002/open.202500364 (PMC12680548; doi:10.1002/open.202500364)
Supplement: Supplementary file 1 — Supplementary Material [file OPEN-14-e202500364-s001.pdf]

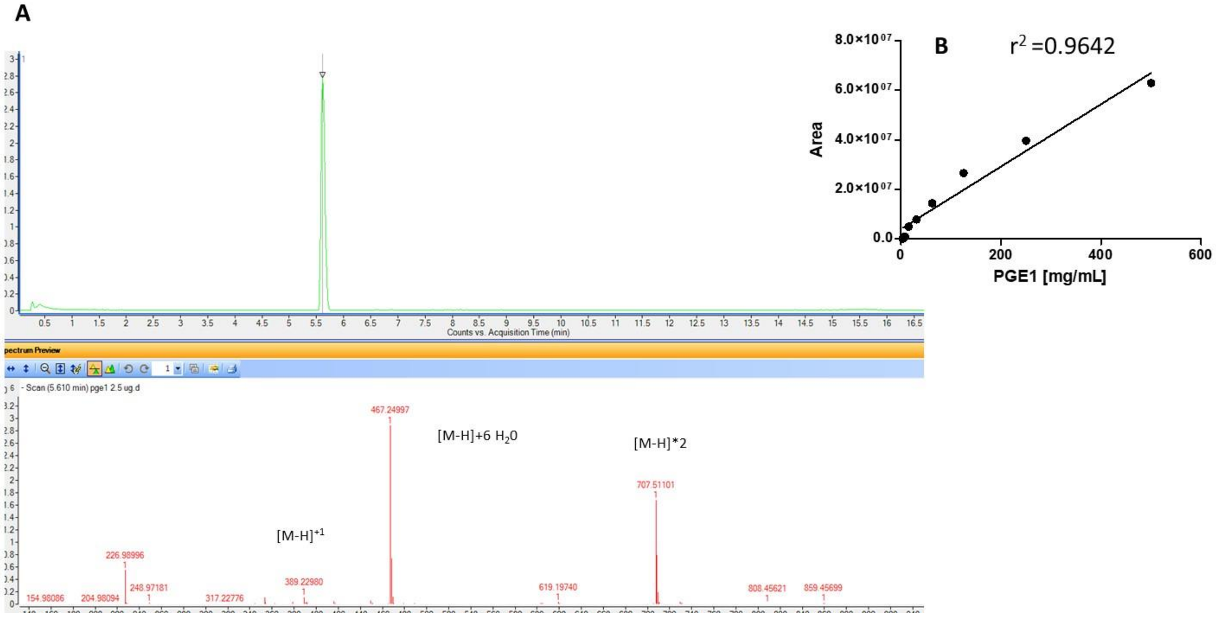

Figure S1: 1) LC-MS of PGE1 standard and its 2) titration curve.

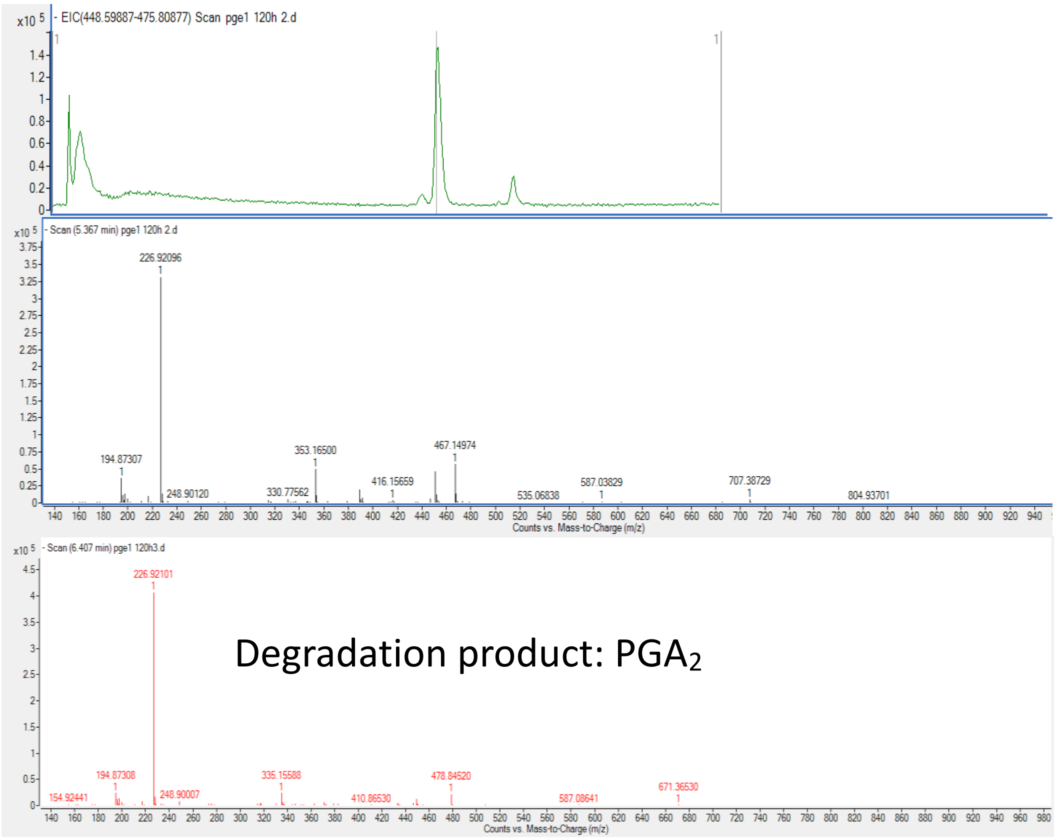

Figure S2) LC-MS of PGE_1 after 120h of release.
